# Gingival Fistula Associated With Pulmonary Nocardiosis

**DOI:** 10.1002/rcr2.70789

**Published:** 2026-10-20

**Authors:** Keigo Uchimura, Sumitaka Kitajima, Makoto Hirai, Teruaki Ishida

**Affiliations:** ^1^ Department of Respiratory Medicine Kasukabe Medical Center Kasukabe Saitama Japan; ^2^ Department of Thoracic Surgery Kasukabe Medical Center Kasukabe Saitama Japan

**Keywords:** bronchoscopy, gingival fistula, nocardiosis, oral manifestation, pulmonary nocardiosis

## Abstract

A 71‐year‐old man receiving prednisolone was diagnosed with pulmonary nocardiosis caused by *Nocardia veterana* and presented with a painless gingival fistula without an apparent odontogenic source. The fistula resolved with antimicrobial therapy alone, suggesting a possible association with nocardiosis and highlighting an unusual oral manifestation.

## Graphical abstract

A 71‐year‐old man receiving prednisolone was diagnosed with pulmonary nocardiosis caused by *Nocardia veterana* and presented with a painless gingival fistula without an apparent odontogenic source. The fistula resolved with antimicrobial therapy alone, suggesting a possible association with nocardiosis and highlighting an unusual oral manifestation of pulmonary nocardiosis.

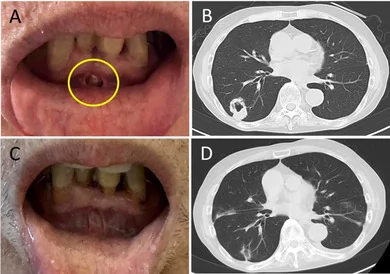

A 71‐year‐old man presented with a painless gingival fistula (Figure 1A, circle), cough, and sputum production. His medical history included chronic heart failure, hypertension, and retroperitoneal fibrosis, for which he was receiving prednisolone (30 mg/day). Chest computed tomography revealed a 3‐cm cavitary lesion in the right lower lobe (Figure 1B). Bronchial washing fluid culture grew *Nocardia* spp., and 16S rRNA gene analysis identified *Nocardia veterana* . He was diagnosed with pulmonary nocardiosis. Dental examination and imaging revealed no apparent odontogenic source. Treatment with trimethoprim‐sulfamethoxazole for 1 week followed by minocycline for 3 weeks resulted in improvement of both the pulmonary lesion and the gingival fistula without dental intervention (Figure 1C,D). Although oral manifestations of nocardiosis have been reported in patients with pulmonary nocardiosis [1, 2], gingival fistula has not been well described in this context. The absence of an odontogenic source and improvement of the fistula with antimicrobial therapy alone suggest a possible association with Nocardia infection. This case highlights gingival fistula as an unusual oral manifestation of pulmonary nocardiosis in an immunocompromised patient.

**FIGURE 1:**
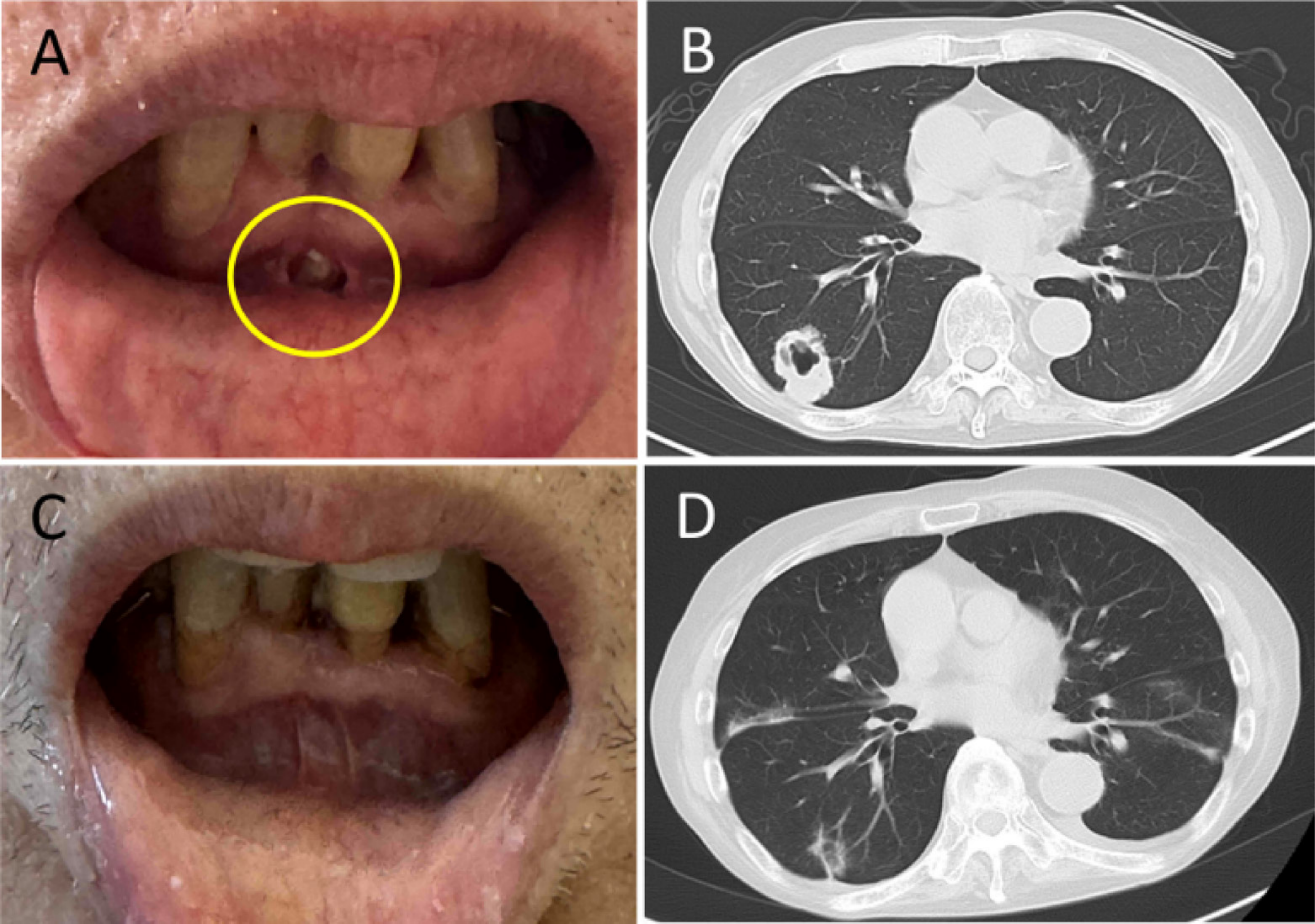
Oral findings and chest computed tomography: (A) Gingival fistula on the lower gingiva before treatment (circle). (B) A 3‐cm cavitary lesion in the right lower lobe before treatment. (C) Improvement of the gingival fistula after antimicrobial treatment. (D) Regression of the right lower lobe cavitary lesion after antimicrobial treatment.

## Author Contributions

**Keigo Uchimura:** writing – review and editing, writing – original draft, data curation, supervision. **Sumitaka Kitajima:** writing – original draft, writing – review and editing, data curation. **Makoto Hirai:** writing – review and editing. **Teruaki Ishida:** writing – review and editing.

## Funding

The authors have nothing to report.

## Disclosure

The authors declared that no Artificial Intelligence (AI)‐Assisted Technology was used for this manuscript.

## Consent

The authors declare that written informed consent was obtained for the publication of this manuscript and accompanying images using the consent form provided by the Journal.

## Conflicts of Interest

The authors declare no conflicts of interest.

## Data Availability

Data sharing not applicable to this article as no datasets were generated or analysed during the current study.
